# Editorial: Training and performance in swimming

**DOI:** 10.3389/fphys.2024.1402543

**Published:** 2024-04-09

**Authors:** Rodrigo Zacca, Sabrina Demarie, Pedro Morouço

**Affiliations:** ^1^ Research Center in Physical Activity, Health and Leisure (CIAFEL), Faculty of Sports, University of Porto (FADEUP), Porto, Portugal; ^2^ Laboratory for Integrative and Translational Research in Population Health (ITR), Porto, Portugal; ^3^ Department of Movement, Human and Health Sciences, University of Rome “Foro Italico”, Rome, Italy; ^4^ ESECS, Polytechnic University of Leiria, Leiria, Portugal; ^5^ CIDESD, Research Center in Sports Sciences, Health Sciences and Human Development, Covilhã, Portugal

**Keywords:** exercice, athletic performance, health, swimming, physical fitness, performance analysis, exercise physiology, aquatic sports

Swimming is part of the Olympic programme since 1896. Water Polo became the first team sport in the Games in 1900, and Diving made its appearance in 1904. Eighty years later, in 1984, Synchronized Swimming joined the Olympics, while Marathon Swimming (10-km race for men and women) was included in 2008 (www.worldaquatics.com/about). In parallel, research in physiology applied to swimming emerged in the beginning of the 20th century, trying to answer several questions that have arisen over the years, particularly focusing on locomotion and its contribution to health and athletic performance (Liljestrand and Stenström, 1920; Zacca et al., 2020; Demarie et al., 2022).

Although so-called “filtered evidence” such as systematic reviews and meta-analyses evaluate and synthesize the literature, they draw from the base of the pyramid, specifically randomized controlled trials - RCTs, cohort studies, and case reports. Narrative reviews, when authored by “Experts” in the field, can also offer valuable insights and contributions. However, we contend that studies at the base of the pyramid, particularly RCTs, cohort studies, and case reports, should be more esteemed than they currently are by scientific, academic, and general population communities. Indeed, considerable physical, intellectual, and scientific effort, even more so in an aquatic environment, as well as financial investment, goes into these studies until they reach the editorial and peer review process. Another important aspect is the challenge of finding volunteers with a high-performance caliber (McKay et al., 2022; Ruiz-Navarro et al., 2023). We need to celebrate quality research in the aquatic environment. You (we) are the true warriors!

This Research Topic aimed to highlight emerging research strategies providing innovative solutions for exercise, health, fitness programs, and sports in aquatic environments. We intended to focus on attracting multifaceted approaches, combining physiology, biomechanics, etc., to address the practical problems that athletes and coaches meet in their daily practices. When we look to the studies from our Research Topic, a strong interest in this area was evident among researchers from Europe, South America, and Oceania. Particularly noteworthy is the enthusiasm from South American countries, as the global community of swimming science will convene in 2026 at the *XV* ^*th*^ International Symposium of Biomechanics and Medicine in Swimming, in Porto Alegre - Brazil, with the general theme “Aquatic Locomotion for All: Health, Exercise, and Sports Performance”. This is especially important, as it gives an opportunity to advance research in this field within this region of the world.

Elite athletes around the world dedicate some time to training at high altitude (Millet and Brocherie, 2020). In teamwork with researchers from Spain and Australia, González-Ravé et al. tracked World and European Championships medalists over a training season in a real-world scenario research, exploring the cumulative effects of multiple altitude camps (21–24 days each) at two high-performance centers (1850 m [Font Romeu, France] and 2,320 m [Sierra Nevada, Spain]).

Swimming equipment serves a dual-purpose during training: it aids in refining technique and facilitates physiological adaptations (Zamparo et al., 2020), while also diversifying the swimming conditions experienced, thereby reducing the monotony of the session. In a collaborative effort between Brazilian and French researchers, De Matos et al. utilized paddles and fins as task constraints in front crawl swimming. Their study aimed to explore the impact of these pieces of equipment on the kinematics, arm stroke efficiency, and coordination patterns during a 50-m all-out test in front crawl.

The respiratory muscles face various challenges that can result in respiratory muscle fatigue and reduced exercise tolerance. The rhythmic contraction of the respiratory muscles drives the movement of air into and out of the lungs, which is intricately linked to the metabolic rate, ensuring the maintenance of blood-gas and acid-base balance (Welch et al., 2019). Researchers from Slovenia and United Kingdom (Moravec et al.) investigated the impact of tumble turns on the occurrence of inspiratory muscle fatigue, comparing it with continuous swimming, and assessed the effects of pre-induced inspiratory muscle fatigue on the kinematic parameters of tumble turns.

Primarily used in neuroimaging to analyze brain imaging data, statistical parametric mapping involves creating spatially extended statistical processes to test hypotheses regarding regionally specific effects (Friston et al., 1991). While it is not directly applied in sports sciences in the same way as in neuroimaging, some analogous applications can be drawn, particularly in the analysis of movement and performance related data. Researchers from Portugal and United Kingdom (Morais et al.) performed a systematic review on the current body of knowledge of using statistical parametric mapping in swimming.

Research Topic concerning the development and training practices of competitive swimming coaches deserve attention by sports sciences community (Crowley et al., 2018). Dalamitros et al. conducted a collaborative study involving researchers from Greece, Spain, Colombia, and Ireland, exploring coaches’ perspectives on their professional development and current training practices.

Often, cross-sectional studies in swimming-related research fail to yield conclusive information regarding the relationships between swimming determinant variables and performance over one or more training seasons. In such cases, longitudinal data become imperative for acquiring comprehensive insights. In this context, researchers from Netherlands (Post et al.) emphasize the significance of elevated initial levels of swim performance and underlying characteristics during late junior age, as well as the capacity to continue improving season-best performances, maximal swimming velocity, turns, and stroke index during the transition from junior to senior levels.

Conducting a cardiopulmonary exercise test (CPET) in a swimming pool presents inherent logistical challenges and limitations due to the underwater environment (Demarie et al., 2001; Zacca et al., 2023). However, researchers from Brazil (Almeida et al.) have offered fresh insights into understanding the correlations between gas exchange responses during CPET in swimming and the concurrent stroke length and stroke rate profiles. This research is valuable to coaches and athletes seeking to enhance their understanding of performance dynamics in aquatic settings.

Despite the scarcity of studies focused on swimming health-related aspects, we are confident that upon reading this Research Topic, you will gain a fresh perspective on training and performance in swimming.
